# Pathogenicity and virulence of African trypanosomes: From laboratory models to clinically relevant hosts

**DOI:** 10.1080/21505594.2022.2150445

**Published:** 2023-01-04

**Authors:** Liam J. Morrison, Pieter C. Steketee, Mabel D. Tettey, Keith R. Matthews

**Affiliations:** a Roslin Institute, Royal (Dick) School of Veterinary Studies, University of Edinburgh, Midlothian, UK; b Institute for Immunology and Infection Research, School of Biological Sciences, University of Edinburgh, Edinburgh, UK

**Keywords:** Trypanosome, human African trypanosomiasis, animal African trypanosomiasis, pathogenicity, virulence

## Abstract

African trypanosomes are vector-borne protozoa, which cause significant human and animal disease across sub-Saharan Africa, and animal disease across Asia and South America. In humans, infection is caused by variants of *Trypanosoma brucei*, and is characterized by varying rate of progression to neurological disease, caused by parasites exiting the vasculature and entering the brain. Animal disease is caused by multiple species of trypanosome, primarily *T. congolense*, *T. vivax,* and *T. brucei*. These trypanosomes also infect multiple species of mammalian host, and this complexity of trypanosome and host diversity is reflected in the spectrum of severity of disease in animal trypanosomiasis, ranging from hyperacute infections associated with mortality to long-term chronic infections, and is also a main reason why designing interventions for animal trypanosomiasis is so challenging. In this review, we will provide an overview of the current understanding of trypanosome determinants of infection progression and severity, covering laboratory models of disease, as well as human and livestock disease. We will also highlight gaps in knowledge and capabilities, which represent opportunities to both further our fundamental understanding of how trypanosomes cause disease, as well as facilitating the development of the novel interventions that are so badly needed to reduce the burden of disease caused by these important pathogens.

## Introduction

African trypanosomes are protozoan parasites, transmitted either cyclically by tsetse flies or mechanically by other biting flies. Several species infect a range of mammals and cause disease, impacting upon both animal and human health. Animal disease is caused by multiple species, with *T. congolense*, *T. vivax* and *T. brucei* the main pathogens of cattle, sheep, goats, equids, and wild animals in sub-Saharan Africa, *T. simiae* and *T. suis* infecting pigs in the same region, and *T. brucei evansi* and *T. vivax* infecting cattle, equids, camels, and Asian buffalo across North Africa, Asia (*T. b. evansi*) and South America (*T. b. evansi* and *T. vivax*). *T. brucei equiperdum* causes a venereally transmitted form of trypanosomiasis in horses and donkeys, mostly in sub-Saharan Africa. Variants of *T. brucei*, *T. b. gambiense* and *T. b. rhodesiense*, also cause human infections and disease in sub-Saharan Africa. The economic and health impact of these pathogens is collectively enormous, with Animal Trypanosomiasis (AT) remaining widespread and causing millions of infections and deaths per year [1–3]. There has been substantial progress in combating human African trypanosomiasis (HAT) in recent decades, in particular for *T. b. gambiense*, with an elimination program in place that aims to remove *T. b. gambiense* HAT as a disease of public health importance by 2030, an objective that seems achievable from recent progress [4]. However, the methods used to control *T. b. gambiense* HAT (active case detection) will not eliminate *T. b. rhodesiense* HAT, due to the truly zoonotic nature and large animal reservoir of the latter pathogen [5]. This outline serves to illustrate the point that trypanosomiasis is caused by a wide diversity of species or variants – and indeed AT can be caused by concurrent infections of multiple species. The genetic diversity within this complex of organisms has begun to be much better understood in the post-genomic era, which has underlined that the species are not only genetically divergent, but that there is also substantial genetic diversity within species (e.g. *T. congolense* Savannah, Forest and Kilifi subtypes). This inevitably means that genetic diversity translates to phenotypic diversity, and this includes virulence.

**Figure 1.**
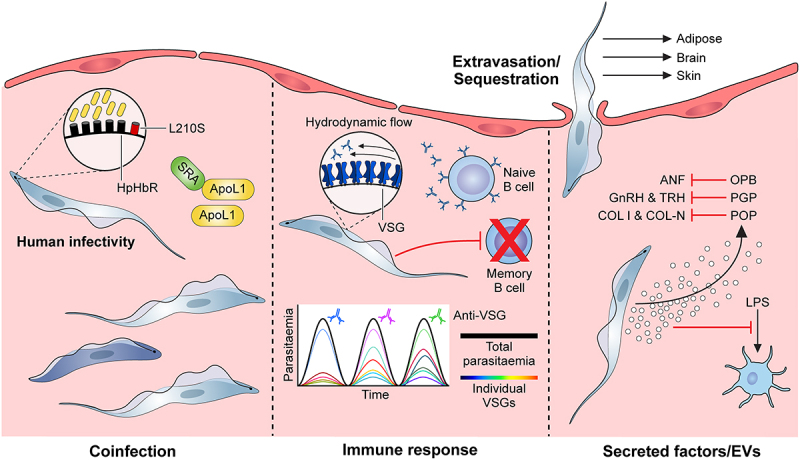
Overview of virulence in African trypanosomes. (from left to right); **Human infectivity**: ApoL1 is the main component of human trypanosome lytic factor (TLF), a high-density lipoprotein subclass that confers protection against animal-infective trypanosomes through parasite lysis. The human-infective trypanosome species, *T. b. rhodesiense* and *T. b. gambiense*, have evolved mechanisms to evade ApoL1-mediated lysis, strongly influencing virulence in human hosts. For example, *T. b. rhodesiense* can express SRA, a protein that neutralises ApoL1 through direct interaction. Another mechanism is reduced ApoL1 uptake via an L210S mutation in the haptoglobin-haemoglobin receptor (HpHbr) that inactivates it. **Coinfection**: Infection with multiple species and/or strains can lead to multiple virulence phenotypes as described. For example, the presence of a less virulent strain can suppress the pathology associated with a more virulent strain of the same species in a coinfection setting. In addition, coinfection of multiple trypanosome species can impact differentiation dynamics. **Immune response**: The interaction of trypanosomes and the host immune response can greatly impact virulence phenotypes. Antigenic variation is undoubtedly a paradigm of trypanosome biology. Hydrodynamic flow of VSGs across the cell surface sweep bound antibodies to the cell posterior, where they are degraded following endocytosis. Furthermore, trypanosomes regularly switch the identity of the expressed VSG, leading to waves of parasitaemia with host antibodies eventually raised to the dominant VSG in the parasite population. A further parasite virulence phenotype associated with the host immune response is the ablation of B cell memory via killing of host B cells. **Extravasation/sequestration**: A key symptom of HAT is an ability of *T. brucei* to extravasate and enter extravascular tissues, in particular the brain, adipose tissue and the skin. A related virulence phenotype has also been described in animal-infective trypanosomes, albeit caused by intravascular sequestration rather than extravasation (e.g. strain and tissue specific sequestration in *T. congolense*). **Secreted factors/EVs**: Trypanosomes release a significant amount of metabolites, proteins and vesicles into the host environment, several of which have been characterised. In particular, virulence associated with secreted peptidases has been established, with oligopeptidase B (targeting atrial natriuretic factor), type 1 proglutamyl peptidase (targeting gonadotropin-releasing hormone and thyrotropin-releasing hormone) and prolyl oligpeptidase (type I and native collagen) all targeting host effectors. As-of-yet unidentified parasite-derived secretome components also target the maturation of host LPS-induced dendritic cells. Abbreviations: ApoL1: apolipoprotein L1; HpHbr: haptoglobin-haemoglobin receptor; SRA: serum resistance-associated protein; VSG: variant surface glycoprotein; OPB: oligopeptidase B; ANF: atrial natriuretic factor; PGP: proglutamyl peptidase; GnRH: gonadotropin-releasing hormone; TRH: thyrotropin-releasing hormone; POP: prolyl oligpeptidase; COL I: type I collagen; COL-N: native collagen; LPS: lipopolysaccharide; EVs: extracellular vesicles. Inset graph in immune response panel adapted from [6].

What do we mean by virulence in trypanosome infections? Virulence can be a very loosely used term in trypanosome literature, often applied to simple phenotypes such as parasite growth rate (including *in vitro*), but it is also used to refer to more complex traits such as host-specific infectivity or vector transmissibility. In reality, many of these phenotypes interact to determine the virulence of a trypanosome. However, virulence is also clearly an outcome of the interaction(s) of the trypanosome with the host, and host factors (for example, host species) can also shape and influence the virulence of trypanosomes in multiple ways. In this article, we define virulence as the ability to cause disease in the recipient host, i.e. the more virulent a trypanosome is, the more severe the disease is in the mammalian host. In this context, we aim to describe what is currently known about the spectrum of virulence diversity in trypanosomes, the variety of mechanisms that underpin virulence in trypanosomes, and the virulence factors that have thus far been identified in trypanosomes (see Figure 1 for overview). Additionally, we will outline the important current gaps in knowledge, and consider the opportunities that recent research presents for advancing understanding in this area. Finally, we will propose priorities for research on trypanosome virulence going forward.

## Evidence of virulence diversity of field isolates

### Human trypanosomiasis

Disease caused by trypanosomiasis in humans is characterized by two stages. The first (the hemolymphatic stage or stage 1) is initiated by the deposition of trypanosomes into the skin by tsetse bite, from where parasites disseminate, initially via the lymphatics, to spread throughout the vascular system. This stage is typified by fever and lymphadenopathy, alongside malaise, weakness, and headaches. The second stage occurs when the parasites invade the brain (meningoencephalitic stage or stage 2) which is associated with motor and sensory dysfunction (including abnormal sleep patterns, giving rise to the colloquial name of sleeping sickness), and if untreated, eventually leads to seizures, coma, and death. The rate of progression from stage 1 to 2 disease is a classical metric of virulence in human infections, and here we will discuss the parasite-driven aspects of this.

As described above, animal and human disease is caused by an assemblage of trypanosome species. The diversity within species can contribute to phenotypic divergence, and in particular can be critically linked to parasite virulence and disease outcome. This is exemplified by the variants (often termed subspecies) of *T. brucei* that are able to infect humans and cause disease; *T. b. rhodesiense*, *T. b gambiense* Group 1 and *T. b. gambiense* Group 2. In fact, the current status of *T. b. gambiense* Group 2 in the area of West Africa from where it was originally described is uncertain [7] – and how these parasites evade lysis by human serum is detailed below. These human infective variants are genetically distinct from each other, and reflect multiple independent emergences of human infectivity as a trait [8]. The genetic divergence also manifests as differences across multiple phenotypes:

- *T. b. gambiense* Group 1 is primarily anthroponotic while *T. b. rhodesiense* and *T. b. gambiense* Group 2 are zoonotic.
- *T. b. gambiense* Group 1 does not undergo sexual recombination and all data suggest this organism reproduces strictly clonally [8–10] – indeed, genomic analysis demonstrated the Meselson effect in this organism whereby there is independent evolution and accumulation of mutations in the homologous chromosomes, which can only occur with long-term absence of sexual recombination [8]. In contrast, evidence from both experimental and population genetics data indicates that *T. b. rhodesiense* and *T. b. gambiense* Group 2 are capable of frequent sexual recombination [8,11–15].
- *T. b. gambiense* Group 1 and *T. b. rhodesiense* require treatment by different drugs, particularly in the meningoencephalitic stage 2 of infection when parasites have entered the brain [16].
- The nature of the disease caused is very different - *T. b. gambiense* Group 1 is classically described as leading to a chronic infection that takes many months to years to culminate in the neurological end stage of infection [17], whereas *T. b. rhodesiense* is usually described as causing infections that tend to be much more acute [18,19], in some instances reaching the critical neurological complications in a matter of days [20].

However, virulence is complex in human-infective trypanosomes, as there is a clear contribution of host genetic variation to disease progression and outcome – this has mostly been defined in *T. b. gambiense* Group 1, where there is evidently a spectrum of disease presentation, including long-term asymptomatic individuals and even apparent self-cure [21]. This difference in outcome has been linked to differential immune response signatures, with high IL10 and low TNFα being associated with an increased risk of developing HAT, whereas increased IL8 was associated with individuals becoming seronegative [22] – in contrast macrophage inhibitory factor (MIF) was shown to be elevated in infected people (HAT patients and latent infections) but to not contribute to pathology [23]. Differential disease outcome has also been shown to be associated with different Apolipoprotein-1 genotypes (see section below on human serum resistance), with patients having G2 alleles of this gene showing improved disease outcome [24], and genome-wide association studies have corroborated this observation [25]. The analysis of host genetic factors is a continuing area of work, and how host genetic diversity may interact with parasite genetic diversity is a remaining question. Indeed, there have been attempts to link *T. b. gambiense* Group 1 genotype to disease presentation in HAT patients [26], but given the remarkable clonality and high levels of homozygosity across multiple isolates [8] it may be that this will prove difficult, and this homogeneity across *T. b. gambiense* Group 1 exemplifies why it may be a good model to explore the human genetic contribution to disease. Having said that, inoculation of *T. b. gambiense* Group 1 isolates into mice deriving from human patients that differed in clinical severity demonstrated that pathology in mice also broadly separated into three categories (“highly pathogenic,” “intermediate pathogenic,” and “low pathogenicity”) that mirrored the pattern of disease severity in the patients they were isolated from [27]. Therefore, there may be a pathogen genetic factor underpinning at least some of the variation in disease outcome observed in *T. b. gambiense* Group 1 infections, perhaps sited in the highly variable subtelomeric regions of the trypanosome genome that are very difficult to assemble and which would not be covered or captured by low-resolution approaches, such as micro- or minisatellites, or by most genome approaches/assemblies. The substantial challenge of incorporating these regions in analysis is evidenced by one study currently being the only genome assembly that has managed to provide a complete picture of *T. brucei* subtelomeric regions [28], despite the organism being the focus of multiple genome sequencing efforts for many years.

The population genetics of *T. b. rhodesiense* is much more complex than that of *T. b. gambiense*, deriving from the fact that *T. b. rhodesiense* is defined by the carrying of a single gene, the serum resistance associated (SRA) gene [29] that confers resistance to the trypanolytic factors in human serum. *T. b. rhodesiense* are therefore essentially variants of *T. brucei* that carry the SRA gene, and the genetic diversity of *T. b. rhodesiense* reflects that of the underlying *T. brucei* population that it is a member of, with clonal expansions occurring when outbreaks occur [8,11,14,30]. As a result of *T. b. rhodesiense* being genetically diverse, there is unsurprisingly also evidence for variation in virulence within *T. b. rhodesiense* in human cases [31]. This is perhaps best characterized by studies in Uganda and Malawi, where it was demonstrated that there were differing population dynamics of *T. b. rhodesiense*, with semi-stable clonal lineages in Uganda and frequent mating in Malawi [14]. These differences likely correlate with their varying transmission intensity and population dynamics resulting in different levels of interactions and mating with the underlying non-human infective *T. b. brucei* population. This also correlated with differing clinical presentations, there being a more chronic form of disease in Malawi and acute disease in Uganda [32]. Within Uganda, with the increased power to test associations afforded by the expansion of clonal lineages, it was possible to link genetically distinct *T. b. rhodesiense* isolated from different disease foci with differing severity of disease presentation [33,34], with circulating IFNγ levels correlating with progression to the neurological stage of disease [33]. In a separate study, genomic analysis was carried out on Ugandan *T. b. rhodesiense* isolates deriving from human patients with differing clinical presentation. This analysis suggested that the genetic divergence of these pathogenically distinct isolates to some extent derived from introgression from West African *T. b. brucei*, with a region on chromosome 8 originating from West African *T. b. brucei* containing a gene(s) whose alleles underpin the virulence differences observed between the isolates [35]. Interestingly, the virulence differences between these *T. b. rhodesiense* isolates variants also was recapitulated to some extent in murine infection models, with isolates of the strain that presented with more severe human disease (Z310) resulting in infections with substantially higher parasitemia and more severe symptoms than isolates from the strain derived from milder human infections (B17) [35].

There have been relatively few attempts to directly examine the genetic determinants of parasite-driven differential pathology, exemplified by *T. b. rhodesiense* and *T. b gambiense*, in *T. brucei*. One approach has been to utilize a classical genetics approach, exploiting cloned progeny derived from a genetic cross between two strains of *T. brucei* (TREU927 and STIB247) that cause very different severity of infection in mice [36]. Analysis of the inheritance pattern of induced pathology during infections with progeny in mice identified a locus on chromosome 3 that was linked to differential organomegaly (enlarged liver and spleen) [37]. While the causative gene(s) in this locus remain to be identified, the phenotype observed involved differential arginase expression and alternative macrophage activation [36], reminiscent of the parasite-induced arginase-mediated reduction of NO synthesis and consequent liver damage by *T. brucei* kinesin heavy chain 1 (TbKHC1 – see section below for further details) [38]. With improved sequencing technologies enabling assembly and annotation of the previously difficult to assemble and highly repetitive subtelomeric regions [28], in which many trypanosome virulence factors are located, it may be timely to revisit the utility of genetic crosses and exploit these resources as routes to identifying virulence factors that may be important for disease.

### Animal trypanosomiasis

While HAT is caused by different variants of *T. brucei*, Animal Trypanosomiasis (AT) is caused by multiple species of trypanosome [39], which are very genetically divergent indeed. These differences are such that traits defined in *T. brucei* as paradigms of African trypanosome biology, such as reliance upon high-rate glucose metabolism in the mammalian host, or the mechanistic utilization of variant surface glycoproteins (VSGs) in antigenic variation, have been shown to either not hold (reliance upon glucose metabolism is much reduced in *T. congolense* [40]) or be achieved by very distinct mechanisms (the VSG content and family structure indicate that antigenic variation is achieved in *T. congolense* and *T. vivax* by different mechanisms to *T. brucei* [41–45]). This means that animal disease is caused by a much greater spectrum of trypanosome genetic diversity, making it a very broad disease in terms of clinical severity, duration and presentation [46]. This complexity is one reason why developing interventions such as drugs and vaccines against AT has been and remains such a challenge, as any product needs to be effective against multiple divergent pathogen species.

Typically in ruminants (cattle, sheep and goats) trypanosome infection manifests as a chronic wasting disease, with intermittent pyrexia, lymphadenopathy, weight loss and reduction in activity [47]. Anemia is a consistent clinical sign in ruminants, and can be used as a diagnostic indicator of infection [48] - albeit with the caveat that several co-endemic pathogens also cause anemia. As well as reduced production in terms of weight loss, trypanosome infections also result in reduced milk yield and fertility [49–51], with abortions in some cases [52,53]. In contrast to the human disease, neurological symptoms are not a common feature of ruminant infections, although seem to be reported more frequently in *T. vivax* infections [54,55]. Donkeys and horses are also commonly infected with trypanosomes [56–58]; in these hosts neurological disease is much more common, with data indicating that *T. brucei* (including *T. b. evansi* and *T. b. equiperdum*) may be implicated in being the primary causative agent of neurological complications [59,60]. Asian or water buffalo (*Bubalus bubalis*) are an important livestock species across Asia, as well as increasingly in South America, and the impact of *T. b. evansi* (as well as *T. vivax* in S. America) upon buffalo is important and currently underappreciated, where it causes a similar chronic production disease to that seen in cattle [46], with occasional outbreaks of high mortality [61]. *T. suis*, *T. simiae* and *T. godfreyi* all either only or preferentially infect pigs, causing either acute or chronic presentation, which is seemingly dependent upon age of infection [62–65]. The extent of the impact of the latter species (as well as other species such as *T. simiae* Tsavo) on livestock has been poorly documented, partially due to lack of molecular tools to enable detection, but also due to a relative paucity of research focus. Similarly, the diversity of trypanosomes identified through molecular screening of tsetse is clearly greater than that of the traditionally described livestock pathogens *T. brucei*, *T. congolense,* and *T. vivax*, (for example the recently described “*T. vivax*-like” species [66,67], but the role that such trypanosomes play in causing disease in livestock is currently unclear.

This brief outline of pathology in animal trypanosomiasis [covered in more detail elsewhere - [46,47,68] serves to illustrate the enormous complexity of the disease, and this can make identifying the contribution of parasite virulence to clinical severity difficult to ascertain – even before considering the added complication and contribution of coinfections (see section below) and host susceptibility/tolerance. However, it is clear that some of the variation in clinical presentation does derive from the parasite genotype. *T. congolense* groups broadly into three genetically distinct subtypes, Forest, Kilifi, and Savannah [39,44,69], and evidence indicates that there is frequent genetic exchange in the field resulting in substantial genetic diversity, at least within *T. congolense* Savannah [70–72], and furthermore suggestion that *T. congolense* Savannah and Forest may hybridize [72]. It has been demonstrated that field isolates representative of the three subtypes differed in virulence in cattle infections – a Savannah isolate (Sam 28.1) causing more severe disease (higher parasitemia, lower packed cell volume and eventually death) than Forest (Dind.3.1) or Kilifi (K60.1A) subtypes, which either showed minimal clinical signs compared to control animals (Kilifi) or even apparent self-cure (or inability to detect parasites in the blood) in all five animals infected with Forest after 95 days of infection (with animals followed for 295 days post-infection) [73]. This strain-specific pattern of virulence was mirrored in mice infections, with Sam 28.1 giving rise to lethal infections that lasted less than a week, whereas Dind.3.1 and K60.1A produced chronic infections with low parasitemia and low mortality rate (one death in each group of seven mice over 130 days of infection) [74]. While the caveat of these studies is that there was a single isolate per subtype, the observations would fit with the increased representation of *T. congolense* Savannah isolates in AT field studies in the literature [39] – the expansion of locations across the African subcontinent where *T. congolense* Kilifi (and to a lesser extent Forest) has been detected has coincided with the advent of more sensitive molecular diagnostics, and this suggests that these subtypes may be reasonably widespread but either cause limited severe disease or present with very low parasitemia in livestock, and are therefore picked up less frequently in surveillance efforts. However, there are really very significant knowledge gaps around *T. congolense* Kilifi and Forest, including the extent of the role they play in livestock disease, and these subtypes certainly warrant further investigation.

Virulence variation within *T. congolense* Savannah has also been demonstrated, with 31 field isolates derived from cattle in Zambia being tested by inoculation into two mice each (with all strains used at their fifth or sixth passage from cattle isolation). The isolates grouped into three categories – termed “extremely virulent,” “moderately virulent” or “low virulence,” as determined by parasitemia profile, survival time, prepatent period, and degree of anemia induced [75]. How these virulence categories translate to clinical presentation in cattle is unclear and requires further work, but it is worth noting that highly virulent field strains of *T. congolense* Savannah have been isolated, which reproducibly give rise to very acute infections in cattle, resulting in death in 9–10 days unless treatment is provided [76–78].

*T.vivax* broadly splits into two genetic groupings, East and West African [45,79,80]. Several studies using different (low resolution) genetic markers indicate that South American strains are derived from West African *T. vivax* [81–83]. While the population genomic analysis of Silva Pereira et al. [45] robustly demonstrated grouping of South American *T. vivax* with Ugandan strains, Uganda is closely linked with West Africa (including via trade routes and transfer of livestock) by being on the edge of the Congo basin, and therefore it is possible that Ugandan *T. vivax* may be more representative of West African strains than those from elsewhere in East and Southern Africa (which were unable to be included in the analysis). This is backed up by a study that assessed cross-reactivity of sera from cattle inoculated with strains from West and East Africa, which showed that sera from cattle infected with Ugandan strains cross-reacted with that from cattle infected with West African strains, but not those infected with East African strains [84]. Such data indicate that further genomic analysis is therefore required to fully resolve the continental picture of diversity for *T. vivax*. With respect to sexual recombination influencing genetic diversity, all evidence suggests that, like *T. b. gambiense*, *T. vivax* is clonal and does not undergo sexual recombination [45,85]. A feature of *T. vivax* infections of cattle (as opposed to *T. congolense* and *T. brucei*) is that self-cure is reasonably frequently reported (e.g. [50,86,87]), and this has been suggested to potentially relate to the smaller VSG repertoire of this species [42,45,50] and therefore “exhaustion” of available VSGs during infections, and/or a reduced amount of VSG protein on the cell surface that may result in greater exposure of other invariant antigens to the host immune response. However, while there are reduced levels of cellular VSG gene transcripts compared to *T. brucei* and *T. congolense* [88], evidence that this translates to reduced levels of VSG protein at the cell surface is less clear [50]. There is reported strain-specific virulence in *T. vivax*, perhaps the most notable being a reported hemorrhagic form of *T. vivax* infection that seems be more commonly reported in isolates deriving from East Africa [50,89–92] – this is associated with a hyperacute infection profile with very high and sustained parasitemia, fever, profound anemia and multiple hemorrhages of visceral and mucosal surfaces. The hemorrhagic stage is correlated with thrombocytopenia and dysregulation of the clotting cascade, as well as generation of autoantibodies that bind to and cause lysis of erythrocytes and platelets [93]. Additionally, other highly virulent field strains (without the hemorrhagic presentation) have been isolated that give rise to experimental infections with very acute profile and short duration (9–10 days before rescue treatment is required) [76,77]. However, while we have good evidence for there being parasite-driven differences in virulence in *T. vivax*, a barrier to understanding the parasite factors that contribute to these differences is that very few *T. vivax* strains grow in mice and only one strain has been reproducibly cultured *in vitro* (Y486) [94,95] - although it should be noted that the culture of bloodstream form Y486 has only been successful to a limited extent. This limitation to ruminant *in vivo* experimental work for all but very few strains means that there has not been the ability to either assess translation of variable virulence in the murine model, or functionally assess potential mechanisms *in vitro*.

## Trypanosome interactions with the host immune response

The host immune system and its interaction with the pathogen evidently is a major component of how virulence presents in the infected mammalian host. The details of immunology in trypanosome infections are well covered elsewhere [68,96–98], and in this section, we will aim to focus on aspects of the host immune response that are driven by the parasite (i.e. how parasite virulence influences elements of the host immune system).

The paradigmal trypanosome interaction with the host immune system is antigenic variation. Trypanosomes have developed an incredibly elaborate and extensive system of antigenic variation, which is driven by a large gene family of variant surface glycoproteins (VSGs), one of which is expressed in each cell through a monoallelic expression system that results in the parasite coat being covered in homodimers of the expressed VSG protein. The VSG N-terminal domains are the primary point of contact for the host adaptive immune response, and VSG epitope-specific antibodies are generated that clear parasites expressing the relevant VSG. However, the parasites regularly change the identity of the expressed VSG, meaning that within a population cells emerge that are not susceptible to the VSG-specific antibodies raised against epitopes on the previously expressed VSG. Through a combination of a very large VSG gene repertoire (2,000 in *T. brucei* – approximately 20% of the coding genome) and elaborate recombinatorial processes that massively amplify the potential encoded genetic VSG variation, antigenic variation in trypanosomes is a powerful tool that matches the host ability to generate antibodies, and is key to their ability to establish and maintain long-lasting chronic infections. The intricacies of antigenic variation, particularly in *T. brucei*, have been the subject of much research over many decades, and the mechanistic understanding is highly developed [43,99,100]. While it is very evident that *T. congolense* and *T. vivax* also undergo antigenic variation, the structure and content of the VSG repertoire in these species is very different to that of *T. brucei* [42,101], and the degree of recombination-driven amplification of diversity also appears quite distinct. For example, while *T. brucei* massively multiplies antigenic diversity through recombination between VSGs that belong to one of two subfamilies (a-VSG and b-VSG), evidence suggests that *T. vivax* does not employ recombinational VSG switching [45], with genes in four subfamilies corresponding to 174 phylotypes (where a phylotype is a clade of highly related VSGs based on amino acid alignment). *T. congolense* lies somewhere between, with a repertoire indicating recombination largely occurring within 15 phylotypes split between two subfamilies [44,101]. Currently, it is unclear if these repertoire differences translate to mechanistic differences in terms of how antigenic variation is expressed in *T. congolense* and *T. vivax* [43]. Additionally, the implications of the VSG repertoire differences with respect to host-parasite interactions, such as the putative different effective repertoire sizes, remain to be elucidated.

The structure of the VSG coat and limited presentation of epitopes to the host response are one mechanism of immune evasion, but trypanosomes also elegantly exploit their motility as an immune evasion technique – the motility driven by the flagellum, combined with the free movement of VSGs across the cell surface, results in hydrodynamic pressures at the cell surface effectively sweeping bound antibodies to the cell posterior, where they are engulfed and removed by endocytosis in the flagellar pocket [102]. This provides an extended time window for trypanosomes to switch VSG identity before antigen-specific antibodies reach a concentration threshold that can overcome the hydrodynamic flow effect. Initially described in *T. brucei*, this has since been shown to also occur in *T. congolense* and *T. vivax* [103] – with species-specific differences in motility characteristics postulated to link to the differential infection biology of the parasites, such as extravascular tissue invasion for *T. brucei*, cellular adherence for *T. congolense* and intravascular circulation for *T. vivax*.

The VSG coat structure has long been posited to provide a near insurmountable barrier in terms of targeting the host immune response to underlying and conserved antigen epitopes, via vaccination for example. How this barrier functions as such, given there are invariant proteins whose structure suggested they should protrude above the protective VSG monolayer, has long been debated [104]. However, the generation of the first model of a full *T. brucei* VSG structure provided insight that the C-terminal domain of the VSG is likely to be remarkably conformationally flexible, sufficiently so to enable VSGs to possibly shield invariant surface proteins [105]. This is supported by efforts that have targeted invariant antigens in vaccination efforts providing at best partial protection [106,107]. Strategies have been implemented to try and bypass this structural barrier, such as using single-chain camel-derived nanobodies against invariant antigens [108]. Overall, this strategy has also met with limited success, although some protective effect was demonstrated. However, recent data has demonstrated that vulnerabilities can be identified by targeting invariant antigens. Through a process of expressing recombinant versions of proteins predicted to be expressed on the cell surface of *T. vivax*, and immunization and challenge experiments in mice, the extracellular domain of one protein (“invariant flagellum from *T. vivax*,” IFX) resulted in reproducibly sterile protection against rechallenge [109]. These remarkable data provide proof of principle that vaccination using surface-expressed proteins may be achievable, after decades of skepticism. The localization of IFX, between the flagellum and cell body, suggests it may play a role in flagellum structure or function, and this may provide a reason as to why it represents a vulnerability for the parasite, as due to its location it may not be subject to hydrodynamic clearance of bound antibodies. Whether this vulnerability also applies to *T. congolense* and *T. brucei* awaits further study. Additionally, the translation of successful immunization against IFX from the mouse model to a clinically relevant host species (goats) has been tried in pilot vaccination and challenge experiments, but did not result in protection [109]. Therefore, significant hurdles clearly remain to be overcome in order to replicate the promising protection observed in mice in disease-relevant hosts such as cattle.

The host antibody response is clearly important in clearance of trypanosomes during infection [110]. Debate continues about the role and efficacy of particular antibody isotypes; for example, the key isotype that conferred protection against *T. vivax* in IFX vaccinations studies was shown to be IgG2a [109], but recent data demonstrated that activation-induced cytidine deaminase (AID)-deficient mice, which are incapable of somatic hypermutation and therefore cannot generate IgG antibodies, were more efficient at clearing challenge with *T. b. evansi* than their wild-type controls through IgM [111] – consistent with previous studies showing the importance of IgM in controlling *T. brucei* infections in mice [112]. Nguyen et al. [111] interestingly hypothesized that the rapid onset of B cell follicle activation and isotype switching to IgG may in fact be driven by the trypanosome, as switching to the lower efficacy IgG would benefit parasite survival. These aspects of antibody response remain to be fully elucidated in the mouse model of *T. brucei*, let alone host species such as cattle, in which the mechanisms of antibody generation are very different and for which the antibodies can have some unique features that may impact upon antigen binding [113,114], and for *T. congolense* and *T. vivax*. If vaccine prospects for AT are to be achieved from candidates such as IFX, clarity on what constitutes an effective antibody response, and how this would be optimally induced, in the eventual host species and against the AT-relevant trypanosome species, will be needed.

Given the key role of antigen-specific antibodies, a notable parasite-driven phenotype is the destruction of host immune memory, with trypanosome infection of mice resulting in ablation of B cell memory via killing of B cells. This included the loss of memory to previously exposed non-trypanosome antigens [115]; this was recently shown to specifically involve the loss of memory B cells from infected animals [116]. This effect has also been shown to occur in mouse infections with *T. congolense* [117], and observed disruptions to splenic architecture including lymphocyte-depleted germinal centers and depletion of splenic B cells in mice infected with *T. vivax* are consistent with the phenotype also occurring in infections with this species [118,119]. While the destruction of B cells has not been formally described in cattle infections, memory loss was observed in cattle immunized with irradiated *T. brucei*, infected with *T. congolense* and then re-challenged with homologous irradiated *T. brucei*, with the memory response against *T. brucei* being impaired in three of the five cattle [120], suggesting that this process also occurs in cattle. The extent of any parasite-driven B cell destruction in human trypanosome infections is also not clear, although one study has demonstrated reduced anti-measles antibody levels in HAT patients [121]. Both cattle and human data, although scanty, indicate that the phenomenon indeed may occur, but the extent of B cell memory loss may not be as extensive as in infected mice. The B cell destruction is known to be mediated by host natural killer (NK) cells and is perforin-mediated [122], but the identity of any parasite ligand that may stimulate and drive this interaction is yet to be identified. While the impact of this parasite-driven phenomenon clearly benefits immune escape and survival of trypanosomes within infections, it also has potentially serious implications for the epidemiology of other infectious diseases in endemic areas. The impairment of immune memory in trypanosome infected animals or humans may mean hosts become more permissive for particular coinfections, and as suggested by other authors [96], the trypanosome-mediated destruction of immune memory would in theory also disrupt vaccination-mediated protection, with consequent implications for disease control efforts. This latter suggestion is backed up by several observations of diminished antigen-specific antibody responses in trypanosome-infected Asian buffalo, goats, and cattle to vaccinations ranging from *Pasteurella multocida*, *Bacillus anthracis*, contagious bovine pleuropneumonia to foot and mouth disease virus [96,123–127]. This aspect of trypanosome infection biology deserves further attention, and in particular fuller understanding of the extent of immune memory loss in clinically relevant hosts, as this could be an important factor in both general disease susceptibility and epidemiology, and, for example, if efforts to generate a vaccine against AT bear fruit.

The symptomology of human trypanosomiasis is defined by the ability of *T. brucei* to extravasate and enter extravascular tissues, leading to encephalitis-related clinical signs. The description of adipose- and skin-resident trypanosomes in mouse and human infections with *T. brucei sensu lato* [128–130] has focused attention on this aspect of *T. brucei* infection biology, with its obvious relevance for disease progression, transmission, diagnosis, parasite metabolism, and interactions with the host immune response. Indeed, the extravasation has been shown in a mouse model to be active (i.e. occurs prior to any vascular compromise induced by inflammation) and if the process is blocked by introducing antibodies against host molecules involved in cellular adhesion (E-selectin, P-selectin, ICAM2, CD36, and PECAM1) mouse survival was improved, indicating that extravasation is a key virulence phenotype in *T. brucei* infections [131]. Notably, CD36 was shown to preferentially inhibit extravasation into adipose depots, indicating potential tissue-specific interaction in extravasation. Brain involvement in the mouse model has also been well defined in infections with *T. vivax* and *T. congolense* [132,133]. In the case of *T. congolense*, which binds to endothelial cells and is considered an intravascular parasite [134], brain pathology was associated with trypanosome sequestration in brain vasculature and the consequent immune response; interestingly this effect was strain-specific (*T. congolense* 1/148 caused sequestration and pathology, while IL3000 did not), suggestive of a differentially expressed parasite virulence factor(s). While similar sequestration in cerebral capillaries and sequelae have been observed in cattle experimentally infected with *T. congolense* [135,136], neurological clinical signs associated with *T. congolense* infections are not frequently reported in the field in livestock [18,46]. It is not completely clear whether *T. vivax* readily extravasates or sequesters, and mouse data has either described mainly vascular lesions [119] or used non-invasive bioluminescence imaging techniques that would not discriminate between intravascular and extravascular parasites [132]. While neurological clinical signs have been reported from *T. vivax* livestock infections in the field [54,137], as with *T. congolense* it is also not a frequent clinical presentation. However, clearly a fuller exploration of tissue distribution in all three parasites, and in clinically relevant hosts as well as mice, is needed before the potentially important implications of tissue specificity and adaptation are understood.

The interaction of trypanosomes with the immune response is clearly multifaceted, and we have chosen here to focus on key parasite-driven aspects. The following sections also contain multiple examples of parasite biology whose interaction with the hosts, including with the immune response, also determine virulence and infection outcome. The examples outlined above particularly serve to illustrate gaps in our knowledge – many of these derive from the need to translate findings from either *T. brucei* to *T. congolense* and *T. vivax*, or from *in vitro* or mouse models to clinically relevant hosts.

## Human infectivity

Another defense mechanism elicited by the mammalian host is the presence of apolipoprotein L1 (ApoL1) in normal human serum. ApoL1 is a component of the trypanosome lytic factor-1 and −2 (TLF1 and TLF2) which is a subclass of high-density lipoprotein (HDL) [138–140]. Human ApoL1 lyses exclusively animal infective trypanosomes through the formation of pH-dependent ionic pores in the lysosomal membrane. This causes the inflow of chloride ions from the cytoplasm leading to lysosomal swelling [141–144], and ultimately, parasite death. Permeabilization of the mitochondrial membrane has also been reported [145]. However, the human infective forms of the parasite, *T. b. rhodesiense* and *T. b. gambiense*, are resistant to these TLFs. *T. b rhodesiense* evades ApoL1 lysis by the possession of serum resistance-associated (SRA) protein [146], which neutralizes the ApoL1 toxin by direct interaction. However, some variants of ApoL1, variants G1 and G2, are able to avoid this deactivation resulting in the killing of *T. b. rhodesiense* [142]. These variants are primarily found in African Americans and West Africans [147], potentially contributing to the absence of *T. b. rhodesiense* infections in west Africa, and factor in the spectrum of disease severity in *T. b. rhodesiense* patients (see “Evidence of virulence diversity of field isolates” section), with the G2 allele being associated with less severe disease in a genetic analysis of *T. b. rhodesiense* patients in Malawi [148]. *T. b. gambiense*, consisting of two groups, Group 1 and Group 2, are both resistant to ApoL1 lysis. While *T. b. gambiense* Group 1 stably avoids TLF lysis, *T. b. gambiense* Group 2 shows variable TLF resistance in a way seemingly similar to *T. b. rhodesiense* but which does not involve SRA, and thus, remains to be fully elucidated [149,150]. *T. b. gambiense* Group 1 on the other hand, uses the specific glycoprotein (TgsGP) to inhibit ApoL1-mediated lysosomal damage by membrane stiffening when it interacts with lipids [151]. Other mechanisms employed by *T. b. gambiense* Group 1 to escape ApoL1 killing include reduced sensitivity to ApoL1 by cysteine proteases [152], reduced uptake of ApoL1 due to an L210S substitution in the haptoglobin-hemoglobin receptor, resulting in inactivation [151,153], and increased digestion of ApoL1 [142]. There have been reports of atypical infections of humans with species of trypanosome not normally infective to humans, including *T. lewisi* and *T. b. evansi*, and very rarely *T. b. brucei*, *T. vivax* and *T. congolense* – while sporadic and clearly rare, instances of such infections either are increasing or are being detected more often [154]. While often the basis for human infectivity in such infections has not been able to be fully investigated, *T. b. evansi* infections have been identified to occur both in an individual lacking APoL1 due to null mutations [155], but also recently in a patient with no observable ApoL1 deficiency [156], suggesting there remain aspects yet to be explained in this intensively studied and important host-parasite interaction.

## Parasite metabolism and virulence

Parasite metabolism is crucial to enable generation of sufficient ATP to persist in the host bloodstream. It is well established that African trypanosomes rely on host carbohydrates in the form of glucose for ATP production. However, metabolic enzymes and their products can also impact host gene expression and metabolism in ways that maximize parasite survival, modulate host immunity, and directly contribute to virulence phenotypes. The role of parasite metabolism in mediating host immune responses has been studied in several pathogenic protozoan parasite species, including *Trypanosoma cruzi* and *Leishmania* spp., in addition to African trypanosomes [157–159]. These parasites all release a significant number of proteins and metabolites into their host environment (the former detailed by studies of the secretome [160–162]), although relatively few studies have detailed the impacts of metabolism on host-pathogen dynamics, and thus, virulence, during infection. Nonetheless, there is clear evidence that parasite-derived metabolites and proteins impact host immune responses with implications for parasite virulence [161,163].

Nitric oxide (NO) is a key host effector molecule in the defense against trypanosome infection and NO exhibits cytostatic and cytolytic properties. To counter the effects of NO, Kinesin Heavy Chain (TbKHC)-1 is a protein secreted by *T. brucei* under both *in vitro* and *in vivo* conditions, and has been shown to induce arginase-1 activity in host myeloid cells, even those from uninfected mice [38]. Arginase-1 converts L-arginine to L-ornithine and urea, and its activity leads to reduction in the synthesis of NO. Presumably, increased competition for L-arginine (an important substrate for NO synthesis) leads to this reduction. Indeed, it has previously been shown that L-arginine bioavailability is an important determinant of NO production and parasite killing [164]. Recombinant TbKHC1 was shown to trigger IL-10 and arginase-1 expression mediated by a C-type lectin (SIGN-R1; CD209b) receptor. Importantly, host survival time is significantly prolonged in TbKHC1 KO-infected mice, compared to wild-type controls [38].

TbKHC1 secretion is not the only form of host NO modulation. Earlier work highlighted that soluble VSG (sVSG), a form of VSG released by trypanosomes, modulates host gene expression in macrophages [165]. Importantly, the timing of sVSG exposure in relation to that of IFN-γ is crucial. Whereas IFN- γ exposure followed by sVSG exposure leads to the expression of TNF-α, IL-6, and IL12p40, treatment of macrophages with sVSG prior to IFN-γ led to a reduction in IFN-γ-induced responses, including reduced NO synthase expression and NO secretion [165]. Further work showed that the glycosylinositolphosphate moiety of the sVSG is crucial for these host modulatory effects [165,166].

Metabolism of fatty acids also impacts virulence. In particular, phospholipase A1 (PLA1) activity is thought to correlate with pathogenesis [167] and indeed, PLA1 activity in plasma and tissue fluid from experimentally infected rabbits correlates with parasitemia [168]. It is thought PLA1 (and potentially PLA2) activity is responsible for the severe changes seen in plasma lipids in infected animals, in particular a reduction in phosphocholines (phosphatidylcholine) accompanied by increased levels of choline, indicative of phospholipase action [169,170]. Interestingly, the phospholipase activity from non-pathogenic trypanosome species such as *Trypanosoma lewisi* is relatively low compared to that of pathogenic species, suggesting a correlation between PLA1 action and virulence/pathogenesis [167].

Trypanosomiasis is also associated with significant perturbations in serum/plasma levels of amino acids [170]. In particular, there is depletion of the aromatic amino acids L-tryptophan, L-tyrosine and L-phenylalanine [171–174]. Concomitantly, *T. brucei* excretes biologically relevant levels of aromatic ketoacids, specifically indolepyruvate (IP), hydroxyphenylpyruvate (HPP) and phenylpyruvate (PP) [171,172,175,176]. These aromatic ketoacids are generated through degradation of aromatic amino acids by the cytosolic aspartate aminotransferase (cASAT) [163,177]. This protein, as well as the reactions it catalyzes, are essential to the parasite [163,178], but the products of these reactions possess several important immunomodulatory properties.

The most studied excretory aromatic keto acid, indolepyruvate (IP), has been implicated in several virulence roles [163,179]. IP is a product of L-tryptophan metabolism through cASAT action [163]. Firstly, IP treatment of host cells leads to reduced glycolytic capacity by interfering with the transcription factor hypoxia-inducible factor-1α (HIF-1α) [163]. Furthermore, this study showed that IP inhibits the induction of pro-IL-1β, a potent pro-inflammatory cytokine. More recent work on IP has highlighted the modulation of host eicosanoid production associated with this trypanosome-derived metabolite [179], specifically the downregulation of a class of eicosanoids called prostaglandins (PGs). In this study, Diskin and colleagues further showed that IP acts as a direct inhibitor of cyclooxygenase (COX) activity, an upstream mediator of PG production, and this effect is replicated in human macrophages [179]. Thus, IP is a powerful modulator of host activity, in particular that of the pro-inflammatory and innate immune response to infection.

Whilst the aforementioned studies were in large part carried out in murine trypanosomiasis models, recent evidence shows that the immunomodulatory properties of IP (in addition to HPP) are replicated in primary human dendritic cells [180], with HO-1 induction through Nrf2 activation, suppressed production of pro-inflammatory cytokines, reduced CD4+ T cell activation and modulation of host cell metabolism, including downregulation of glycolytic capacity [180]. To our knowledge, there are no reports on any immunomodulatory effects of PP, another aromatic ketoacid excreted at high levels by African trypanosomes. Unlike IP, PP has no effect on the ability of LPS to induce IL-1β [163], but other roles cannot be ruled out.

In murine models, trypanosome infection is associated with global host metabolic disturbances, including in the bloodstream, but also in other anatomical locations such as the gut and brain [170,181,182]. These changes are the result of both host and parasite metabolism. The main glycolytic end-product from trypanosomes is pyruvate, which accumulates to high levels in the host plasma [170]. There are also increased plasma concentrations of lactate and these are, to an extent, indicative of upregulated glycolysis in host cells [170]. *T. brucei* does not encode lactate dehydrogenase (LDH) [183], and, therefore, cannot generate lactate via fermentation of glucose [184]. However, glucose-derived L-lactate is excreted from *T. brucei* at low levels, likely via methylglyoxal detoxification [183,185]. It should be noted that procyclic form *T. brucei*, as well as bloodstream form *T. lewisi*, also excrete L-lactate, the latter able to do so via lactate dehydrogenase [186,187]. It is currently unknown whether the livestock trypanosomes *T. congolense* and *T. vivax* generate L-lactate via fermentation, although LDH is not annotated in their respective genomes. It is plausible that both host and parasite-derived lactate likely contribute to metabolic acidosis, a significant contributor to pathology. As the disease progresses, the host can enter a ketotic state, characterized by increased levels of D-3-hydroxybutyrate, where lipids are metabolized for energy [170]. This is partially due to competition for the main energy source, glucose [188–190]. Ultimately, hypoglycemia can play a role in host survival [and has been noted in cattle infections, e.g. [54]], and, therefore, parasite glycolytic rates have the potential to impact upon parasite virulence.

Several other important metabolic processes have been shown to impact upon virulence, including proteases such as serine peptidase 2 (ISP2) [191] and the cysteine proteases Cathepsin L and Cathepsin B [192]. In addition, increased levels of O- and N-acetylated glycoproteins have been detected in *T. brucei*-infected plasma, which are likely *T. brucei* derived [193]. Whilst the underlying mechanisms remain to be elucidated, it is clear that these proteins are involved in trypanosome-mediated attenuation of the immune response [191–193]. Finally, *T. brucei*, like other pathogens, exhibits an ability for metabolic mimicry, where *T. brucei* derived inositol phosphate glycans released from GPI anchors are able to affect the host in the same way as insulin, an important hormone for glucose regulation [194].

Recent evidence has revealed that *T. brucei* is capable of invading adipose tissue [130], a site abundant in glycerol. Indeed, *T. brucei* is capable of proliferation in glycerol-based medium [195], and these findings may also contribute to our understanding of trypanosome virulence *in vivo*. Furthermore, imbalances in plasma lipid bioavailability have also been detected in plasma samples derived from experimental model and human infections [196–198].

Whilst the majority of studies on trypanosome metabolism and its impact on virulence have focused on the relevant model for human infection, *T. brucei*, comparatively few studies have investigated the relevant species for Animal Trypanosomiasis - *T. congolense* and *T. vivax*. Recent studies, however, have shown that the former differs from *T. brucei* in key metabolic areas, such as glycolysis and lipid metabolism, and this may impact metabolic phenotypes associated with virulence [40]. For example, it was hypothesized some time ago that free fatty acids released from autolyzing trypanosomes can significantly impact pathogenesis and virulence [199]. The differences in fatty acid metabolism between *T. congolense* and *T. brucei* that have been observed recently could underpin differences in virulence between the species [40]. Furthermore, differences in metabolite uptake and excretion may also play an important role in differing virulence between African trypanosome species, but these are as yet unstudied.

Whilst the genetic basis of differential virulence in livestock trypanosomes has not been elucidated, there is clear evidence that strain-dependent variation in virulence exists in *T. congolense* [73–75], and it is likely that differential metabolism underlies at least some aspects of this variation. *T. vivax* is unique amongst African trypanosomes in that it encodes a proline racemase not found in *T. brucei* or *T. congolense* [200]. This enzyme was subsequently shown to be a potent B-cell mitogen and thus, can be considered a virulence factor underpinning hypergammaglobulinemia, a symptom observed during acute stages of *T. vivax* infection in mice [200]. Furthermore, unlike *T. brucei*, adhesion to host cells is an important aspect of *T. congolense* and *T. vivax* bloodstream form biology, as well as pathogenesis [134,201,202]. In both *T. vivax* and *T. congolense*, it is established that trans-sialidases are involved in host cell attachments, and are also a key mediator of anemia, and thus, virulence [203–206]. Trans-sialidases are both expressed on the parasite surface and secreted into the extracellular environment, and they are responsible for desialylation of red blood cells, leading to erythrophagocytosis and anemia. There is evidence that trypanotolerant N’Dama (African taurine) cattle exhibit reduced anemia compared to susceptible indicine cattle [207], and concomitant evidence that trans-sialidases purified from *T. vivax* desialylated indicine but not African taurine RBCs [203], indicating a correlation between trans-sialidase host-specificity and parasite virulence.

Comparative proteomics analysis of *T. vivax* strains with contrasting virulence revealed differential expression of several metabolic enzymes [208]. In that study, virulence and pathogenesis were interpreted as capacity to multiply and capacity to produce disease/mortality, respectively. Protein expression profiles of two strains (high virulence and moderate pathogenicity vs low virulence and high pathogenicity) were compared. Amongst the significant differentially expressed proteins, there were also important glycolytic enzymes, pyruvate kinase, and glycerol kinase, expressed at higher levels in a *T. vivax* strain eliciting significantly more severe clinical pathogenesis, suggesting that glycolytic metabolism may play a role in driving symptoms [208].

**Figure 2.**
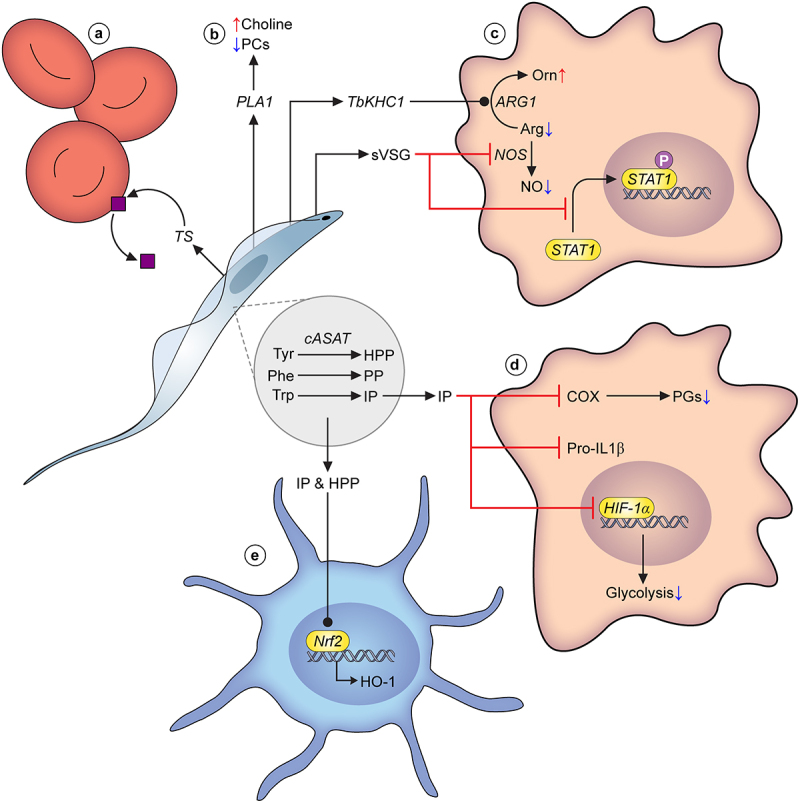
Parasite metabolism and virulence. A) Trans-sialidases released by *T. vivax* cleave sialic acid moieties from glycoproteins on the erythrocyte cell surface, leading to erythrophagocytosis and eventually, anaemia. B) All three species of pathogenic African trypanosomes are known to release phospholipases that degrade phosphocholine-bound lipids. They are considered significant virulence factors, and their action results in a build up of choline in the host bloodstream. C) *T. brucei* secretes multiple factors that modulate macrophage ability to generate nitric oxide (NO), including TbKHC1, and soluble VSG (sVSG). The latter stimulates arginase-1 activity, leading to increased usage of the available arginine pool to generate ornithine, reducing substrate availability for NO production through nitric oxide synthase (NOS). Simultaneously, sVSG has an inhibitory effect on NOS. sVSG also interferes with the phosphorylation of STAT1, an important transcription factor that drives pro-inflammatory responses. D) Parasite amino acid metabolism and its effect on host responses has been studied to some degree in *T. brucei*. In particular the fate of hydroxyphenylpyruvate (HPP), phenylpyruvate (PP) and indolepyruvate (IP), products of cASAT-catalysed conversions of L-tyrosine, L-phenylalanine and L-tryptophan, respectively. IP is a potent modulator of pro-inflammatory responses in macrophages. Firstly, IP interferes with HIF-1α, leading to a reduction in glycolytic capacity of macrophages. Secondly, IP inhibits induction of pro-IL-1, a potent pro-inflammatory cytokine. Finally, more recent work has established that IP is a direct inhibitor of cyclooxygenase (COX), leading to reduced prostaglandin (PG; mediators of inflammation) production. E) Trypanosome-derived IP as well as HPP can impact upon dendritic cells, by stimulating Nrf2-mediated hemeoxygenase-1 (HO-1) induction, again leading to a reduced pro-inflammatory response. Many other metabolic factors are known to be excreted by trypanosomes, but their molecular interactions with the host environment remain to be established, and they are therefore not included in this overview figure.

It is worth noting that *in vivo* experiments focused on dissecting host–pathogen interactions and virulence have been carried out almost exclusively on rodent models in contrast to clinically relevant models such as, in the case of animal trypanosomiasis, cattle [95]. Ruminants such as cattle exhibit markedly divergent blood biochemistry from non-ruminants such as rodents and humans [209], and this has the potential to impact both parasite and host metabolism during infection. For example, ruminant blood contains reduced levels of glucose and substantially increased levels of small volatile fatty acids (e.g. propionic acid and butyric acid) [210–212] compared to human or mouse blood. Thus, given that, as an example, glycolysis is a cornerstone of trypanosome metabolism, host metabolic differences may play an important role in influencing host–pathogen interactions and virulence (Figure 2 summarizes our current understanding of how trypanosome metabolism influences infection severity and outcome).

## Quorum sensing

Although trypanosomes are single-celled parasites, individuals within the population show the ability to act co-operatively to restrict parasite numbers. This has a direct impact on virulence and parasite transmissibility. Virulence is affected because with unlimited population growth, hosts lethally and rapidly succumb [213,214]. Correspondingly, the prolongation of host viability increases the probability of transmission – the essential requirement for any parasitic lifestyle. This is particularly the case for African trypanosomes where transmission is restricted by the poor vectorial capacity and relative scarcity of tsetse flies in comparison to, for example, mosquito vectors for parasites such as *Plasmodium* [215–217]. In addition to the direct consequences of uncontrolled parasite proliferation on host survival, the co-operative behavior of trypanosomes acts to promote transmission by driving the generation of non-proliferative transmission adapted developmental forms of the bloodstream parasites – so-called stumpy forms [218]. These forms, specific to *T. brucei* at least as a morphologically distinct entity, dominate the peak of acute and chronic parasitaemias in experimental infections and predominate in tissue reservoirs such as the skin and adipose tissue when quantitated using the molecular marker defining this form, PAD1 [129,130,219].

Stumpy generation is a quorum sensing phenomenon whereby parasite numbers are detected and responded to by individuals within the population – a characteristic described in many species of social microbe. Evidence of inter-parasite communication controlling the production of stumpy forms was initially provided by the analysis and modeling of parasites in animal infections [220], but definitive evidence emerged with the successful culture of parasites with the developmental competence to generate stumpy forms [221,222]. These are representative of tsetse-transmitted trypanosomes in the field and are termed pleomorphic [223], and are distinguished from so-called monomorphic forms that arise through laboratory passage, or in parasite subspecies that have lost the capacity for tsetse transmission, and are instead spread either by mechanical transmission by other biting flies (*T. b. evansi*) or by venereal transmission between equids (*T. b. equiperdum*) [224,225].

**Figure 3.**
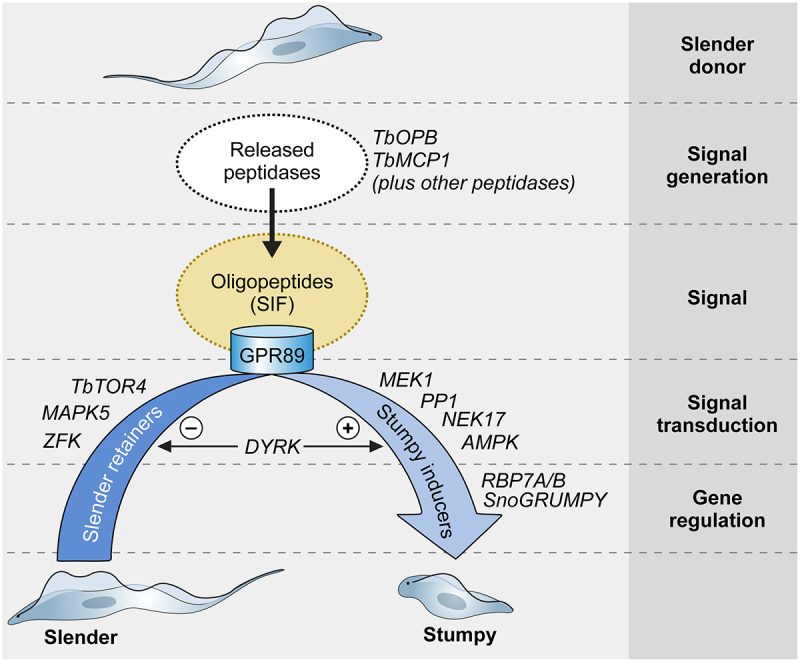
Quorum sensing in *Trypanosoma brucei*. Schematic pathway for the quorum sensing signalling pathway in *Trypanosoma brucei*. Slender form parasites release several peptidases into their environment, with two peptidases, Oligopeptidase B and Metallocarboxypeptidase I being important contributors to the generation of the quorum sensing signal, oligopeptides. Environmental oligopeptides can be transported into recipient parasites by the TbGPR89 surface transporter that is expressed on slender cells but not stumpy forms. In an unknown mechanism, transported oligopeptides stimulate a signal transduction cascade that promotes stumpy formation through the action of gene regulators (RNA binding proteins). Molecules that act to inhibit stumpy formation (slender retainers) are inactivated. At least one kinase, TbDYRK, acts on both control arms, inhibiting slender retainers and promoting stumpy formation. Other molecules, annotated as “Hypothetical proteins” in TryTrypdb (https://tritrypdb.Org/tritrypdb/app) have been identified that control stumpy formation but their positions in the regulatory pathway are unknown.

The molecular details that generate the quorum sensing signal and how this is detected and transduced to effect development in the parasites have been recently unraveled (Figure 3). The signal that induces the parasite to undergo cell cycle arrest and stumpy formation (classically described as an ill-defined “stumpy induction factor”, SIF) is oligopeptides in the environment of the parasite [226]. These are generated by proteolytic enzymes or peptidases that are released by the parasite in the mammalian host, apparently through an unconventional protein secretion pathway [227]. This allows a density-dependent signal to be generated because as parasite numbers increase, the abundance of the released peptidases correspondingly increases and, though their activity in the blood or tissues, produce oligopeptides to activate the developmental signaling pathway [228]. Two peptidases have been found to provide a major contribution to the generation of the QS signal, Oligopeptidase B and metallocarboxypeptidase 1, and the individual or combined deletion of both peptidases by gene knockout increases parasite virulence through reduced stumpy formation [227]. Other parasite-derived peptidases are also likely to contribute, however, complementing the dominant activities of TbOPB and TbMCP1, or pre-processing larger polypeptide substrates so that they can act as substrates for these enzymes, which show specificity for substrates limited in size [229,230]. Host peptidases in the parasite’s environment could also provide a signal to augment stumpy formation; this would not be dependent upon parasite numbers directly, although immune responses to the parasite population may involve proteolytic activities [231]. Although untested, the immune response against the parasite could also contribute to promote quorum sensing if parasite-specific antibodies proximal to the parasite could provide substrates for trypanosome-released peptidases, potentially contributing to the altered stumpy formation of intact versus immunocompromised mice [232]. Importantly, the generation of oligopeptides by parasite-derived peptidases allows stumpy formation at high parasite numbers in the blood and also low parasite numbers where trypanosomes are constrained within tissues such as the skin and adipose, such that local accumulation of their activities and products can occur [226,227]. This resolves the perceived conundrum that stumpy formation in rodents involves large parasite numbers, whereas in livestock hosts the circulating parasite population might be relatively low but stumpy forms are prevalent [233].

The presence of oligopeptidases activates a developmental signaling response. The signal is transported by a surface molecule, TbGPR89, specific to slender cells as the signal-receiving population. Interestingly, not all oligopeptides operate equally effectively, with tripeptides being more active than dipeptides, and with particular amino acid combinations being more effective than others [226] - suggesting a specificity code. The quorum sensing signaling pathway has many components, originally identified via a genome-wide RNAi screen designed to isolate parasites unresponsive to a cell permeable mimic of the quorum sensing signal [234]. These molecules include protein phosphatases and protein kinases as well as RNA binding proteins acting as predicted gene regulators and, more recently, a long non-coding RNA regulator [235]. Several hypothetical proteins of unknown function are also implicated [236]. In combination, these components drive stumpy formation, although an analysis of their respective dependency relationships indicated that the signal transduction pathway was not a simple linear hierarchy [237]. Perhaps more than one signal input contributes to ensure appropriate activation of the developmental response, or perhaps there is regulatory input or feedback from other molecular components, including those not yet uncovered? Molecular inhibitors of stumpy formation have also been identified [238–241], as has at least one molecule that seems to act on both stimulatory and inhibitory arms of the process [242]. This reflects the complexity and stringent regulation of quorum sensing, which is necessary because stumpy formation represents a terminal developmental step unless the parasites are transmitted to tsetse.

The gene expression response to the quorum sensing signal has been analyzed by single-cell RNA sequencing [243]. This identified the trajectory of the transition in terms of gene expression from the slender to the stumpy forms. In particular, parasites were observed to transition in the G1 phase of their cell cycle with no “intermediate form” transcriptome identified, despite the description of these morphologically transitional forms [223]. Analysis of a parasite line defective in quorum sensing through its deletion of a component of the QS signaling pathway identified early transcript-level changes in gene expression as parasite initiate the developmental QS response [243], providing a route to pinpoint the molecular commitment events that define the initiation of the decision to progress toward stumpy formation.

The generation of stumpy forms is a unique innovation to *T. brucei* with limited evidence for morphological development in either *T. congolense* or *T. vivax*. Nonetheless, both these species exhibit density-dependent growth arrest, accumulating as G1-arrested forms at higher parastiaemias [244,245]. In *T. congolense*, gene expression changes that accompany this arrest have been analyzed, which predicts changes in the expression of some surface proteins [246]. The genomes of both *T. congolense* and *T. vivax* also encode orthologues of many of the regulators of quorum sensing identified in *T. brucei*, and at least one of these (TcIL3000.0.19510) can complement a *T. brucei* null mutant for TbHYP2 (Tb927.9.4080) to restore stumpy formation, demonstrating functional equivalence [245]. Thus, despite the absence of morphological development, it appears both *T. congolense* and *T. vivax* exhibit quorum sensing to regulate their virulence in mammalian hosts and, potentially, as an adaptation for tsetse uptake.

## Secreted factors and EVs

The importance of released peptidases and their role in generating the signal that promotes the development transition to stumpy forms has reemphasized that trypanosomes are not passive entities in their hosts but instead behave interactively to support their survival and transmission and, potentially, to contribute to virulence.

Several studies have analyzed the secretome of bloodstream form trypanosomes. Secreted proteases, and *T. brucei* Cathepsin-L (TbCatL) in particular, have been implicated mediating extravasation of *T. brucei* cells via perturbation of intracellular calcium levels in brain endothelial cells [247], and secreted TbCatL has also been shown to induce spontaneous depolarization events in isolated cardiomyocytes, also via perturbation of intracellular calcium levels [248], which may contribute to cardiac pathology observed in human and animal infections. Secreted peptidases have been proposed to be involved in trypanosome pathogenicity by hydrolyzing host hormone peptides, hence affecting their functions [24950^250^, . Other studies have also described peptidases in the blood of infected mice and rodents where they remained catalytically active [251–253]. Various studies have looked at these secreted or released peptidases and their substrates both *in vitro* and *in vivo*. For example, oligopeptidase B (OPB), which cleaves Arg/Lys containing peptides smaller than 30 amino acid residues, is released into the bloodstream of rats infected with trypanosomes. This cleaves available host hormones such as atrial natriuretic factor resulting in hormonal deregulation linked with trypanosome infections [249,253]. Similarly, type 1 pyroglutamyl peptidase (PGP) and prolyl oligopeptidase (POP) are also released into the blood of rats during trypanosome infections and remain catalytically active. PGP cleaves host gonadotropin-releasing hormone (GnRH) and thyrotropin-releasing hormone (TRH) by removing the N-terminal pyroglutamic acid-blocking groups [252] while POP, which hydrolyses Pro/Ala containing substrates at the carboxyl end, hydrolyses type 1 collagen and native collagen [251]. POP has also been demonstrated to hydrolyze substance P, oxytocin, vasopressin, and angiotensin *in vitro* [251]. In all, the expression of peptidases, their secretion/release into the host bloodstream and their activity in the host plasma have been reported in African trypanosomes, with interest in these molecules enhanced with the recent discovery of their involvement in the generation of the quorum-sensing signal for stumpy differentiation [227,228].

Numerous studies have looked at the secretome of the different species of trypanosomes, including in the different life cycle forms [160,161,254,255–258]. These studies have identified proteins that are involved in different functions, including folding and degradation, nucleotide, carbohydrate and amino acid metabolism, protein synthesis, and transport [258]. These proteins may interact with the host’s immune system, and by so doing, contribute to immunopathology and also the survival of the parasites. This was demonstrated by Garzon et al. [257], where they found a reduction in the secretion of host immune molecules and an impairment in the maturation of lipopolysaccharide (LPS)-induced dendritic cells in the presence of *T. b. gambiense* secretome. As highlighted above, among the identified secreted proteins in previous studies were peptidases. Different classes of peptidases are expressed in trypanosome parasites; serine, cysteine, metallopeptidases, threonine, and aspartyl with peptidases from each of these classes being characterized in *T. brucei*.

Exosomes are a subset of extracellular vesicles and are formed from the budding of the late endosomes. They are cup-shaped, approximately 20–100 nm in diameter [259], and are secreted through fusion with the plasma membrane. In a variety of pathogens, exosomal secretion has been proposed to be involved in cell-to-cell communication, cell-to-host communications, and has also been implicated in pathogenicity and cell differentiation [260–262]. Protein release through exosomes has been described in trypanosomes when the secretome of the parasite was analyzed, revealing the release of leaderless proteins as well as exosome associated proteins such as Rab proteins, clathrin heavy chain, enolase, and heat-shock protein 70 [254]. Extracellular vesicular secretion has also been reported in *T. cruzi* [259], and in *Leishmania* it was recently demonstrated that genes encoding drug resistance can be passed between *Leishmania* cells via exosomes [263] – highlighting the potential importance of this phenomenon for multiple phenotypes. Molecules involved in this type of secretion include the components of the ESCRT complex (Endosomal Sorting Complex Required for Transport) [262,264,265].

## Parasite-parasite interactions and coinfections

The extracellular release of factors into their environment open the possibility for coinfecting trypanosome strains and species to interact with one another through collaboration or competition, either directly or indirectly. Different trypanosomes species co-circulate in sub-Saharan Africa, with transmission by the same vector species [266]. As a consequence, coinfections have been frequently reported both in livestock infection and in trapped tsetse flies [267–269]. In several studies, the coinfection between different strains and species have been found to alter the infection dynamics and/or virulence in the host [57]. This was exemplified in concomitant infections of livestock with virulent and less virulent strains of *T congolense* in livestock, where the presence of a less virulent strain suppressed the pathology associated with a more virulent strain [270]. This was not linked to shared antigens or immune cross reactivity between the parasites in the infection, since removing one of the strains with trypanocides before inoculating the second strain eliminated the response. Competitive suppression has also been observed between strains of *T. brucei* in rodent infections, with a more virulent strain being suppressed by a less virulent strain, enabling extended host survival and reduced pathology [271]. These phenomena are reminiscent of the interactions between closely related *Theileria* species in livestock, where the impact of the more pathogenic *Theileria parva* was ameliorated by infection of the less virulent *Theileria mutans*, to the extent that infection with the less virulent species was proposed as a potential anti-virulence approach to disease control, potentially more valuable than anti-parasitic drugs [272]. In many cases, however, the coinfection of different parasite species generates significantly worse outcomes [273,274].

Interactions between strains in coinfection are also observed with respect to quorum-sensing signals. As highlighted earlier, *T. congolense* does not generate morphologically stumpy forms but the parasite exhibits arrest in G1 in a density-dependent manner [245]. To explore whether the parasites could detect the QS signals, mice with *T. congolense* infections were superinfected with *T. brucei* and the ability of the latter to generate stumpy forms compared with mice infected with *T. brucei* alone. The *T. brucei* in the coinfection were found to accelerate their stumpy formation reflective of the overall parasite load, rather than the contribution of *T. brucei* to the infection alone. Furthermore, the response of the parasites was dependent on their intact QS signaling pathway since null mutants for a component of the signaling path proliferated in the coinfection setting regardless of the existing *T. congolense* parasites [245]. Overall, this demonstrated that *T. brucei* responds to the presence of *T. congolense* and that this is mediated via a *T. congolense* derived QS signal, although whether there was a signal from *T. brucei* to *T. congolense* could not be explored in the absence of suitable molecular markers for transmission stages in the latter species.

These experiments established that different trypanosome strains and species can detect and respond to one another in a coinfection and that this can alter their potential for virulence and transmission. Such interactions in the field have the potential to select for evolutionary strategies that provide an advantage in a coinfection scenario for parasites that reduce their sensitivity to the QS signal, or occupy niches where they are less susceptible to manipulation by a coinfecting strain [218]. As discussed elsewhere, there are also potential implications for therapeutic strategies [275] or with species-specific vaccination [109], since this has the potential to perturb a coinfection equilibrium to allow the emergence of parasites less sensitive to QS signals and so more likely to be virulent when in a monoinfection setting.

## Future perspectives: identification of gaps, priorities, and opportunities

In this article, we have aimed to summarize current knowledge with respect to parasite factors that influence the severity of clinical signs and disease outcome in the mammalian host. The complexity of disease caused by trypanosomes, with the involvement of multiple host and multiple trypanosome species, means that this is inevitably an ambitious undertaking, and covers multiple aspects of trypanosome infection biology. However, the advances in the last decades by many researchers mean that we now have an impressively detailed understanding of trypanosome factors that influence host-parasite interactions. This is particularly true for *T. brucei*, and for infections in mice, where the understanding is particularly advanced – the detailed knowledge we now have of the differentiation process from long slender to stumpy life cycle stages being a prime example. In recent years, efforts in *T. brucei* have also begun to significantly shift toward working with pleomorphic, differentiation-competent strains (e.g. *T. brucei* EATRO 1125 AnTat 1.1) and away from the heavily laboratory-adapted monomorphic Lister 427, a move that will provide data more relevant to field infections. However, the science reviewed in this article highlights that we have identified relatively few virulence factors responsible for even well characterized phenotypes (see Table 1), and has also highlighted areas where knowledge in general is much less developed. These gaps in understanding represent opportunities for the trypanosome research community going forward, and in the following section we aim to outline research needs and opportunities.

**Table 1.** Virulence phenotypes and state of current knowledge.

Phenotype	Mechanistic details	Virulence factor	References
Human infectivity	Evasion of trypanosome cell lysis by preventing lytic activity of human Apolipoprotein-1.	SRA (*T. b. rhodesiense*) TgsGP, TbHpHbR, KIFC1, V-ATPase-a, -c, -F & -H, V-ATPase assembly factors,Tb9297.10.12940, Tb927.9.8000 (*T. b. gambiense* Group 1) Unknown gene, probably located in bloodstream expression site (*T. b. gambiense* Group 2)	[149],[276,^277^ [29,146,151]
Tissue damage	Modulation of NO production via influencing arginase expression, leading to liver injury and early mortality. (*T. brucei;* mice)	TbKHC1	[38]
	Strain-specific organomegaly, linked to arginase expression and alternative macrophage activation. (*T. brucei*, mice)	Locus on *T. brucei* chromosome 3 – gene unknown	[3637^37^,
	Cardiomyocyte depolarization and cardiac arrhythmias via parasite-induced perturbation of host cell Ca^2+^ signaling. (*T. brucei*; rats)	TbCatL	[24878^278^,
Hypergammaglobulinemia	B-cell mitogenesis in response to virulence factors released by the parasite. (*T. vivax*)	Proline racemase	[200]
Anaemia	Desialylation of erythrocytes leading to erythrophagocytosis. (*T. vivax* & *T. congolense*)	Trans-sialidases	[204–206]
Antigenic variation	Evasion of host antigen-specific antibody response by sequential switching of identity of the expressed Variant Surface Glycoprotein. (*T. brucei*, *T. congolense*, *T. vivax*; all mammalian hosts)	VSG	[42–45,99]
Host B cell destruction	Ablation of splenic B cells, Including memory B cells, leading to loss of adaptive immune memory, including to non-trypanosome antigens. (*T. brucei*, *T. congolense*, *T. vivax*; mice, probably cattle & humans)	Unknown	[115–118,122]
Trypanosome differentiation	*T. brucei* – Genes identified in a genome wide RNAi screen for resistance to chemical activators of the stumpy formation pathway in vitro, cAMP or AMP. The listed genes have been confirmed to be involved in stumpy formation *in vivo* (mice). Negative regulator of stumpy formation Negative regulator of stumpy formation Negative regulator of stumpy formation Long non coding RNA regulator of stumpy formation positioned between Tb927.10.12090 and Tb927.10.12100. Oligopeptide transporter for the quorum sensing signal Peptidases contributing to the generation of the quorum sensing signal	Tb927.4.3620/30/40 (PP1) Tb927.10.5930/40/50 (NEK17) Tb927.2.2720 (MEK) Tb927.10.15020 (DyrK) Tb927.11.6600 (Hyp 1) Tb927.9.4080 (Hyp2) Tb927.10.12090 (RBP7A) Tb927.10.12100 (RBP7B) Tb927.2.4020 (Nedd8 activating enzyme) Tb927.3.4560 (AMPKa) TbTOR4 MAPK5 ZFK SnoGRUMPY TbGPR89 TbOPB, TbMCP1	[142,234,237] [238,239,241] [235] [226] [227]
	*T. congolense* – functional orthologue of Tb927.9.4080 (45% identity, 58% similarity).	TcIL3000.0.19510	[246]
	*T. vivax* – not known		
Skin colonisation	Invasion (& metabolic adaptation?) and establishment of skin resident populations. (*T. brucei;* mice & humans)	Unknown	[128,129]
Adipose tissue colonisation	Invasion & metabolic adaptation to adipose tissue. (*T. brucei;* mice)	Unknown	[130]
Endothelial cell adherence & sequestration	Strain-specific cerebral pathology linked to differential sequestration in brain capillaries. (*T. congolense;* mice) Parasite-induced migration across endothelial barriers by perturbation of host cell Ca^2+^ signaling. (*T. brucei*)	Unknown TbCatL	[133] [202,247]
Metabolic suppression of macrophage activity/response	Indolepyruvate impacting upon glycolytic capacity through HIF-1α interference and inhibition of pro-IL-1β. Inhibition of cyclooxygenase and downstream prostaglandin production via indolepyruvate. (*T. brucei*)	Indole pyruvate (derived from cASAT; Tb927.10.3660; predicted syntenic orthologues: TcIL3000_10_2990, TvY486_1003700	[163]
Metabolic modulation of dendritic cell response	Activation of Nrf2 and HO-1 induction impacting upon glycolytic capacity, leading to reduced production of pro-inflammatory cytokines and CD4+ T cell activation. (*T. brucei*)	Indole pyruvate & hydroxyphenylpyruvate (derived from cASAT; Tb927.10.3660; predicted syntenic orthologues: TcIL3000_10_2990, TvY486_1003700)	[163]

While the ability to work with *T. brucei* advances at a spectacular pace, with examples such as precise and scalable gene silencing and editing techniques that facilitate high throughput phenotypic screens and highly detailed functional analysis [234,279–281], the rate of progress in terms of advancement in knowledge, data, and capabilities lags significantly behind for *T. congolense* and *T. vivax*. Partly, this is due to the sheer difference in scale in terms of amount of research investment into these pathogens, as evidenced by the stark disparity in the number of research outputs on *T. congolense* and *T. vivax* compared to *T. brucei* over the past decades [282]. However, there has been a refocusing on *T. congolense* in recent years that has seen the generation of foundational datasets and capabilities [40,44,72,133,283–285], and combined with renewed interest from funders in AT, this is contributing to a revival of research on this organism. Although not to the same extent, there are also tools and resources available for *T. vivax* that enable work on this pathogen that was not previously possible [45,88,94], which is already leading to notable, paradigm-challenging studies [109]. However, there are key basic capabilities where improvement would be transformative. The ability to culture bloodstream form *T. congolense* and *T. vivax in vitro* is still limited, with a very small number of strains for *T. congolense* having been successfully adapted to *in vitro* culture, and only one for *T. vivax* – with a requirement in *T. vivax* for mammalian feeder cells for mid- to long-term culture. Development of media formulations that supported axenic growth of multiple strains for both species would be a substantial breakthrough that would accelerate meaningful functional studies, and provide the ability to dissect the genetic diversity present in both species. Additionally, while genetic modification is clearly possible in both species [94,283], in *T. vivax* this is currently restricted to the epimastigote life cycle stage, and requires differentiation through metacyclics to bloodstream forms to obtain the relevant mammalian life cycle stage. The ability to directly genetically modify *T. vivax* bloodstream form cells would be a major step forward, albeit this may depend on the prior development of an appropriate *in vitro* culturing medium. It is clear that the multiple fundamental differences between these three species mean that if such basic capabilities are improved, there are many opportunities for identifying novel and important aspects of trypanosome cell and infection biology.

As is evident from many of the advances outlined in this article, the mouse model has been and remains hugely influential and useful in providing key insights into the infection biology of African trypanosomes. The mouse model provides experimental tractability and scalability that can make it an incredibly powerful experimental tool. However, ultimately for most traits there is a need or desire to assess translation to the clinically relevant host model. It should be stressed that this is not always simply a matter of assessing clinical relevance, but that it can also be because analyzing a trait in such hosts provides genuine scientific insight and interest. While this is clearly a challenge with the human disease in terms of both ethical reasons and the small and reducing number of clinical cases (although the extravascular skin populations of *T. brucei* are a recent example of laboratory observations proving important and useful in HAT [128,129]), for AT there is substantial scope for analysis in clinically relevant hosts such as cattle. Several facilities are now available that enable experimental infection of cattle, and there are multiple phenotypes observed either *in vitro* or in the mouse model that it would be of significant interest to translate to the bovine model – whether for reasons of host size and scale, physiology, bovine-specific aspects of the immune response, or to demonstrate potential clinical relevance. Example phenotypes where the interest in this translation to the cow are obvious include antigenic variation (where modelling has indicated host size is likely to play a substantive role in VSG-population dynamics [286]), parasite-mediated B cell memory loss [115], cell adherence and tissue-specific sequestration [133], coinfection and interaction between trypanosome species [245], and the validation of vaccine candidates [109]. However, this is to name but a few phenotypes where analysis in the bovine host would prove informative; it is abundantly clear that many aspects of our understanding of trypanosome infection biology would greatly benefit from assessment in the bovine or other clinically relevant host species.

Similar to increased research on the clinically relevant hosts, there is equally a lot of value to be gained from more assessment of the extent to which laboratory findings are recapitulated in the field. Likewise, findings in the field also have the obvious potential to stimulate meaningful laboratory and experimental work, and there could perhaps be better integration of laboratory and field approaches across many aspects of trypanosome biology. The tissue distribution of trypanosomes in the skin and adipose, and the findings that have resulted from the original studies describing these phenomena [129,130], is a prime example of the mutually beneficial advances that can be gained across basic and applied approaches from combining field and laboratory experimental work. But as noted above, there are multiple aspects of trypanosome biology where integration of field approaches with laboratory approaches would enhance our understanding – for example, the increasing interest in coinfections, where experimental approaches provide the ability to control for multiple confounders and gain mechanistic insights, but properly designed field approaches enable the deconvolution of the interaction of trypanosomes (or mechanism) with host, pathogen, and environmental factors.

There also remain some fundamental gaps in our field knowledge of trypanosome virulence. For example, almost all *T. congolense* studies (laboratory or field) focus on *T. congolense* Savannah. We know astonishingly little about the other subtypes of *T. congolense*, Forest and Kilifi, other than that they are detected in cattle and wildlife across sub-Saharan Africa, a very few assessments of relative virulence of selected strains in cattle and mice, and the generation of a limited amount of genome data (for Forest). But the extent of the disease caused in cattle and other livestock attributed to these subtypes, and the intricacies of their epidemiology (e.g. tsetse transmission, interaction with other trypanosome species and other subtypes of *T. congolense*) are questions that are ripe for answering – similar questions pertain to several of the understudied trypanosomes, such as *T. simiae*, *T. suis* and *T. vivax*-like. This lack of knowledge feeds into some of the capability challenges outlined above (e.g. limited capacity for *in vitro* culture), but is likely also exacerbated by confirmation bias in many field study designs (i.e. targeted surveillance will only detect what is tested for). Improved diagnostics, and in particular the increasing power of sequence capture or enrichment approaches, should enable a better disentangling of such questions.

In summary, reflecting the complexity of disease caused by multiple and genetically distinct trypanosome species that infect a diversity of host species, virulence in trypanosomes is complicated and multifactorial. While we have made substantial progress in some areas and have a good understanding of virulence factors and how they exert their effect, particularly with respect to *T. brucei*, there remain substantial gaps in knowledge. However, these represent opportunities going forward to further our understanding of how these fascinating and important pathogens interact with the mammalian host and cause disease. This has the potential to reveal yet more scientifically novel aspects of trypanosome biology, as well as lead to the design of novel interventions that are still sorely lacking for both human and animal trypanosomiasis.
